# Development of a prediction model with serum tumor markers to assess tumor metastasis in lung cancer

**DOI:** 10.1002/cam4.3184

**Published:** 2020-06-14

**Authors:** Jiasi Wang, Yanpeng Chu, Jie Li, Fanwei Zeng, Min Wu, Tingjie Wang, Liangli Sun, Qianlai Chen, Pingxi Wang, Xiuqin Zhang, Fanxin Zeng

**Affiliations:** ^1^ Department of Clinical laboratory Dazhou Central Hospital Dazhou Sichuan China; ^2^ Department of Cardiology Peking University First Hospital Beijing China; ^3^ Department of Clinical Research Center Dazhou Central Hospital Dazhou Sichuan China; ^4^ Huaxi MR Research Center Department of Radiology West China Hospital Sichuan University Chengdu China; ^5^ Institute of Molecular Medicine Peking University Beijing China

**Keywords:** cut‐off value, decision tree model, lung cancer, metastasis assessment, nomogram model, tumor markers

## Abstract

**Background:**

This study aimed to explore the possibility of serum tumor markers (TMs) combinations in assessing tumor metastasis in patients with lung cancer.

**Methods:**

We performed a retrospective analysis of 541 patients diagnosed with lung cancer between January 2016 and December 2017 at the Pneumology Department of Dazhou Central Hospital. Serum carcinoembryonic antigen (CEA), carbohydrate antigen (CA)125, CA153, CA199, CA724, cytokeratin 19 fragment (CYFRA), and neuron‐specific enolase (NSE) levels were quantified in each patient at the time of lung cancer diagnosis. Metastasis was confirmed by computed tomography, and/or positron emission tomography, and/or surgery or other necessary methods. Receiver operating characteristic (ROC) curves and calibration curves were used to evaluate the performance of the model.

**Results:**

Of the 541 patients eligible for final analysis, 253 were detected with metastasis and 288 were detected without metastasis. Compared with those in nonmetastatic patients, the serum CEA, CA125, CA199, CA153, CYFRA, and NSE levels were notably higher in metastatic patients (*P* < .05). The ROC curve demonstrated that the CEA‐CA125‐CA199‐CA153‐CYFRA‐NSE‐CA724 combination based on the cut‐off value had an optimal area under the curve and specificity in assessing tumor metastasis. The decision tree model is a convenient and valuable tool for guiding the appropriate application of our model to assess metastasis in lung cancer patients.

**Conclusions:**

Our study suggested that the nomogram of the regression model is valuable for assessing tumor metastasis in newly diagnosed lung cancer patients before traditional standard methods are used. These findings could aid in the evaluation of metastasis in the clinic.

## Graphical abstract

The study developed a predictive model integrated with age, gender, and seven serum tumor markers (based on cut‐off value), which was efficient and reliable for assessing tumor metastasis before traditional standard methods are applied in highly suspected lung cancer patients. The predictive model combined with the decision tree model could provide some positive suggestions in the clinic.

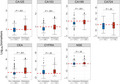

## 1 INTRODUCTION

Lung cancer is one of the most fatal cancers and is the leading cause of cancer‐related death.^1^ Recently, an estimation by the American Cancer Society suggested that in 2019, lung cancer will still be the leading cause of death related to cancers, and the number of new lung cancer cases will be ranked the second highest among all types of cancers in the United States.^2^ Lung cancer often cannot be diagnosed until an advanced stage is reached.^3^ The survival rate of lung cancer patients remains low, with a 5‐year survival rate varying from 6% to 18% depending on gender and region.^4^ It has been reported that recurrence and metastasis significantly increase the risk of death of lung cancer patients.^5^ The 5‐year overall survival rate for non–small cell lung cancer (NSCLC) is 68% in patients with stage IB disease but less than 10% in patients with stage IVA‐IVB disease.^6^ Patients with extensive‐stage small‐cell lung cancer (SCLC) have a median survival of 10‐12 months.^7^ Thus, the identification of metastasis has important guiding value for the selection of clinical treatment regimens for newly diagnosed lung cancer patients and their subsequent prognosis. A previous study has reported that in NSCLC patients who have no more than five metastases, appropriate therapies can result in 13% of patients having no progression in 3 years, even in stage IV patients who can benefit from radical therapy.^8^

Compared with imageological examinations, such as computed tomography (CT), chest X‐ray, positron emission tomography‐CT (PET‐CT), and magnetic resonance imaging (MRI), which cannot be performed frequently and are expensive, blood‐based biomarker tests are economically acceptable and can be assayed easily and quickly. Thus, they have the potential to greatly improve the efficiency of assessment. Carcinoembryonic antigen (CEA), cytokeratin 19 fragment (CYFRA), neuron‐specific enolase (NSE), and the carbohydrate antigen (CA) series, such as CA125, CA153, CA199, and CA724, are traditional and common tests used to assist in the diagnosis of tumors but lack solid evidence.^9^, ^10^, ^11^ Some recent studies showed that the combination of these biomarkers in lung cancer could improve diagnosis and monitor the treatment effect.^12^, ^13^, ^14^ However, the use of these biomarkers to assess the metastasis of lung cancer has not been reported.

In this study, we retrospectively analyzed the relationship between different combinations of biomarkers (CEA, CA125, CA153, CA199, CA724, CYFRA, and NSE) and tumor metastasis in newly diagnosed lung cancer patients and investigated their clinical value in the diagnosis of lung cancer metastasis.

## 2 MATERIALS AND METHODS

### 2.1 Study cohort

Total 2635 patients between January 2016 and December 2017 at the Pneumology Department of Dazhou Central Hospital with pulmonary bronchoscopy records were potentially included in the study. The exclusion criteria were as follows: (a) patients diagnosed with nonmalignant lung diseases (n = 2061); (b) patients diagnosed with lung cancer, but lack histological diagnosis (n = 29); and (c) patients who lacked all values of serum tumor markers (TMs) (n = 4). Finally, 541 patients pathological diagnosed with lung cancer and had no other malignant diseases or malignant diseases history were enrolled in the study (Figure S1). Clinical information, including gender, age, histological diagnosis, tumor size, and serum TMs, was retrospectively obtained from electronic medical records. The study was approved by the Medical Ethics Review Board of Dazhou Central Hospital. The Medical Ethics Review Board waived the need for informed consent from the participants in this study.

### 2.2 Tumor biomarker assays

The serum CEA, CA125, CA153, CA199, CA724, CYFRA, and NSE levels were detected at the admission according to the manufacturer's instructions. Their standard reference range, upper reference limit (URL) value, and detected protocols are shown in Appendix S1.

### 2.3 Reference standard

In our study, all patients underwent fiberoptic bronchoscopy biopsy at the initial time of cancer diagnosis after hospitalization. Lung cancer was determined by pathological diagnosis according to the clinical standards. The specimens for pathological diagnosis were from fiberoptic bronchoscopy, percutaneous lung biopsy, or surgical resection. The histological subtypes of lung cancer were diagnosed by pathologists according to the pathological morphology and immunohistochemistry.

Metastasis detection in the study was based on the identification of lung cancer and combined imaging evidence and patients’ clinical characteristics (if necessary, combined with pathological examination and/or expression levels of TM, such as partial lymph node metastasis confirmed by lymph node dissection, presence of pathological evidence of pleural effusion and pericardial effusion, and high expression of CA125 in pleural effusion), as well as the timeliness of imaging examination, that is, the metastases we confirmed were within the first hospitalization period after admission (no more than 1 month). CT, MRI, and fluorodeoxyglucose (FDG) PET‐CT scans were used as the imaging modalities for the assessment of metastasis. To avoid bias, any evidence metastasis was confirmed by 2 professionals with more than 10 years of experience and at least 2 senior doctors with more than 10 years of clinical practice.

The size of the tumor was determined by the senior doctors in the imaging department. Tumor size measurements were made with accuracy to mm using a professional length measurement tool on software (INFINITT Healthcare Co, Ltd) from three planes (coronal plane, transverse plane, and sagittal plane). In the analysis, the longest tumor diameter in the largest transverse plane was selected.

### 2.4 Definition of groups

The metastasis group: patients diagnosed with lung cancer, and with detected metastases within the first hospitalization period (no more than 1 month), were included.

The nonmetastasis group: patients with lung cancer but without lymph node, intrapulmonary, or any other metastasis detected within the first hospitalization period, and patients with metastasis detected beyond the first hospitalization period were included.

### 2.5 Statistical analysis

The results are expressed as numbers, medians (with interquartile ranges), or proportions. The Wilcoxon test was used to compare the differences in the levels of the TMs, and *t* test was used to compare the differences in age. A chi‐square test was used to compare the proportions between groups. The independence test of categorical variables was based on the chi‐square independence test or the Mantel‐Haenszel test. Receiver operating characteristic (ROC) curves were calculated for logistic regressions based on a single biomarker or multiple biomarkers (and/or combined gender and age) and stepwise regressions in which the mode of stepwise search was used. R (version: R 3.4.3 for Windows (×64); https://www.r‐project.org/) was used for statistical analysis. A *P*< .05 was considered statistically significant. See Appendix S1 for the unabridged version of Section 2.5.

## 3 RESULTS

### 3.1 Participant characteristics

The finally enrolled 541 patients included 421 males and 120 females, with a median age of 63 years (range from 26 to 83 years). Of the 541 patients diagnosed with lung cancer, 20 patients (3.8%) could not be accurately defined by histology due to the mixed type of squamous cell carcinoma and adenocarcinoma, while the other 521 patients had a definite histological diagnosis (Table 1). Of all patients with identified histology, 119 had SCLC (119/541, 21.9%) and 402 had NSCLC (402/541, 74.3%).

**TABLE 1:** Clinical characteristics and tumor markers in all participants

	Stratified by metastasis		*P*‐value
	Metastasis (n = 253)	Nonmetastasis (n = 288)	
Gender = Female (%)	71 (28.1)	49 (17.0)	.003
Age (mean [SD])	60.21 (9.37)	62.62 (8.96)	.002
Pathological typing (%)			.221^a^
NSCLC	185 (34.2)	217 (40.1)	
SCLC	63 (11.6)	56 (10.3)	
Other	5 (1.0)	15 (2.8)	
CEA, ng/mL	7.38 [3.43, 27.63]	3.41 [2.44, 5.47]	<.001
CYFRA, ng/mL	5.87 [3.33, 9.83]	4.78 [2.82, 8.46]	.039
NSE, ng/mL	19.92 [13.29, 29.85]	15.63 [11.23, 24.36]	.004
CA125, U/mL	60.83 [24.99, 151.35]	28.47 [14.85, 62.08]	<.001
CA153, U/mL	19.38 [13.41, 39.53]	14.07 [10.34, 21.56]	<.001
CA199, U/mL	14.85 [7.35, 44.75]	10.60 [6.44, 17.39]	<.001
CA724, U/mL	4.86 [1.88, 14.76]	3.84 [1.48, 11.02]	.183

Note

Data are given as n (%) or median (IQR) unless otherwise noted.

Abbreviations: CA125, carbohydrate antigen 125; CA153, carbohydrate antigen 153; CA199, carbohydrate antigen 199; CA724, carbohydrate antigen 724; CEA, carcinoembryonic antigen; CYFRA, cytokeratin‐19 fragment; NSCLC, non–small cell lung cancer; NSE, neuron‐specific enolase; SCLC, small cell lung cancer.

^a^ Means patients with Pathology typing = ‘Other’ was excluded.

### 3.2 The URL vs the cut‐off value of individual biomarkers

**FIGURE 1:**
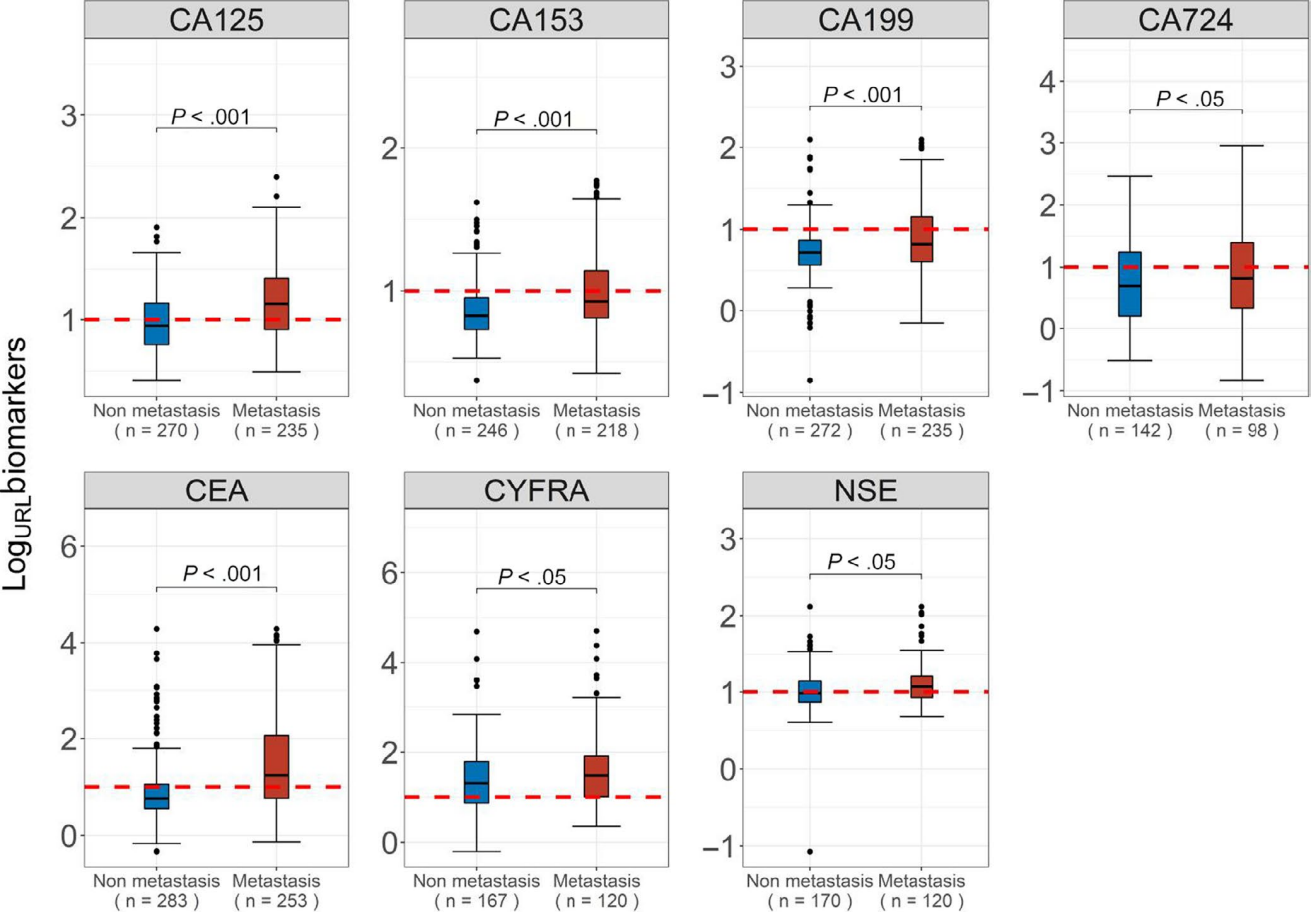
Distribution of levels of tumor markers in metastasis and nonmetastasis lung cancer. CA125, carbohydrate antigen 125 (U/mL); CA153, carbohydrate antigen 153 (U/mL); CA199, carbohydrate antigen 199 (U/mL); CA724, carbohydrate antigen 724 (U/mL); CEA, carcinoembryonic antigen (ng/mL); CYFRA, cytokeratin‐19 fragment (ng/mL); NSE, neuron‐specific enolase (ng/mL); URL, Upper Reference Limit of individual biomarker. Red line indicates the URL

Lung cancer patients with metastasis showed significant differences in the gender proportion (*P* = .003; Table 1) and age (*P* = .002; Table 1) compared with patients without metastasis (Table 1). Patients in the metastasis group had a higher female proportion (28.1% vs 17%) and were younger (60.21 vs 62.62 years) than those in the nonmetastasis group. In the metastasis group, the CEA, CYFRA, NSE, CA125, CA153, and CA199 levels were obviously higher than those in the nonmetastasis group (Table 1; Figure 1).

The URL of the individual biomarkers could not effectively assess metastasis (Figure 1), which suggested that metastatic assessment with the URL of a single biomarker may cause deviations. The cut‐off values of the single biomarkers calculated by ROC curves of each TM are shown in Table S1.

**FIGURE 2:**
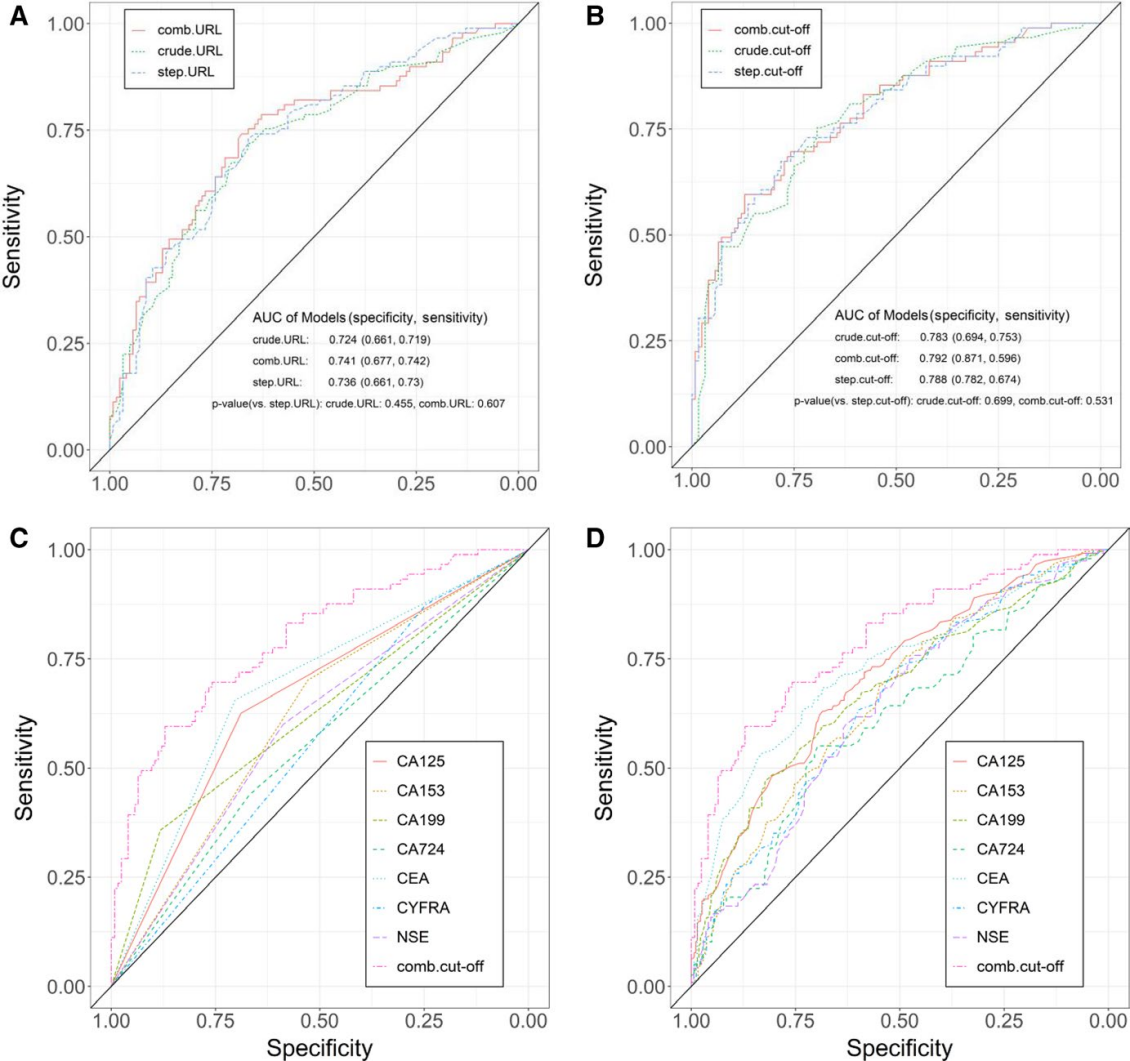
Receiver operating characteristic curves of the clinical model compared with the logistic regression models. A, Logistic regression models based on the upper reference limit of the tumor markers. B, Logistic regression models based on the cut‐off value of the tumor markers. C, Adjusted without the gender and age factors according to the cut‐off value. D, Adjusted with the gender and age factors according to the cut‐off value

To investigate the performance value of single TMs in assessing tumor metastasis, we compared the ROC curves derived from the URL value vs those derived from the cut‐off value. Compared with the areas under the curve (AUCs) by the URL values, the AUCs by the cut‐off values for all TMs were increased. Moreover, the specificity for some TMs was improved. CA125 showed a significant difference in the ROC curves for the cut‐off value (*P* < .01; Table 2). To further investigate the performance of the URL value vs the cut‐off value in individual TMs, we compared the differences in the true negative rate (TNR), false negative rate (FNR), false positive rate (FPR), and true positive rate (TPR) in different grouping methods. The TNR of CA153 showed a decrease (Figure S2A), while the TPR of CA153 showed a considerable increase for the cut‐off value (70.2%) compared with the URL value (37.6%; Figure 2D). The TNR, FNR, FPR, and TPR of other TMs had a certain degree of optimization (Figure S2A‐D). These results suggested that a single TM could not accurately assess metastasis.

**TABLE 2:** Performance of individual tumor markers (grouped by upper reference limit vs Grouped by cut‐off value, individual biomarker)

	Grouped by upper reference limit			Grouped by cut‐off value			*P*‐value
	AUC (95% CI)	Specificity (95% CI)	Sensitivity (95% CI)	AUC (95% CI)	Specificity (95% CI)	Sensitivity (95% CI)	
CA125	0.614 (0.571‐0.656)	0.559 (0.500‐0.619)	0.668 (0.609‐0.728)	0.657 (0.616‐0.699)	0.689 (0.633‐0.744)	0.626 (0.562‐0.689)	<.01
CA153	0.593 (0.552‐0.633)	0.809 (0.760‐0.858)	0.376 (0.312‐0.440)	0.615 (0.572‐0.659)	0.528 (0.467‐0.589)	0.702 (0.638‐0.761)	.29
CA199	0.613 (0.577‐0.649)	0.886 (0.846‐0.923)	0.340 (0.281‐0.400)	0.620 (0.584‐0.656)	0.882 (0.842‐0.919)	0.357 (0.298‐0.417)	.15
CA724	0.543 (0.480‐0.607)	0.648 (0.570‐0.725)	0.439 (0.347‐0.541)	0.554 (0.491‐0.617)	0.669 (0.592‐0.739)	0.439 (0.337‐0.541)	.08
CEA	0.671 (0.631‐0.710)	0.721 (0.668‐0.770)	0.621 (0.557‐0.684)	0.680 (0.640‐0.719)	0.707 (0.650‐0.760)	0.656 (0.597‐0.711)	.20
CYFRA	0.534 (0.481‐0.586)	0.317 (0.246‐0.389)	0.750 (0.667‐0.825)	0.560 (0.516‐0.604)	0.246 (0.180‐0.311)	0.875 (0.817‐0.933)	.14
NSE	0.577 (0.520‐0.635)	0.529 (0.453‐0.600)	0.625 (0.542‐0.708)	0.594 (0.537‐0.652)	0.588 (0.512‐0.659)	0.600 (0.516‐0.692)	.14

Abbreviations: AUC, area under the curve; CI, confidence interval.

### 3.3 Tumor marker values stratified by tumor size and pathology subtype

Tumor size was divided into four groups: ≤3.0, 3.1‐5.0, 5.1‐7.0, and >7.0 cm in our study. When the tumor size was in the ≤3.0, 3.1‐5.0, and 5.1‐7.0 cm groups, CA125, CA153, and CEA showed significantly higher levels in the metastasis group than in the nonmetastasis group. CA199 showed significantly higher levels in the metastasis group than in the nonmetastasis group when the tumor size was 3.1‐5.0 cm. CA724 showed significantly higher levels in the metastasis group than in the nonmetastasis group when the tumor size was 5.1‐7.0 cm. Cytokeratin 19 fragment and NSE showed no significant difference between the metastasis and nonmetastasis groups in all tumor size distributions. When the tumor size was >7.0 cm, no significant difference in all TMs values was observed between the metastasis group and the nonmetastasis group (Table S2). The proportion of participants with abnormal TM values stratified by tumor size is shown in Figure S3A,B. When stratified by tumor size according to the cut‐off value, the proportion of patients with abnormal CA199 and CA153 values was significantly different between the metastasis and nonmetastasis groups with tumor sizes of 3.1‐5.0 cm compared with that stratified by tumor size according to the URL. Additionally, the proportion of participants with abnormal CA125 values was significantly different between the metastasis and nonmetastasis groups in those with tumor sizes ≤3.0 and >7.0 cm when stratified by the nodule size according to the cut‐off value (Figure S3A,B). The Cochran‐Mantel‐Haenszel test showed that the difference in the number distribution of each TM between the metastasis group and nonmetastasis group was not affected by tumor size (*M* ^2^ = 0.708, *P* = .87; Table S3).

In NSCLC, CA125, CA153, CA199, CEA, CYFRA, and NSE were notably higher in the metastasis group than in the nonmetastasis group (Table S4). In SCLC, only CA199 and CEA had significant differences between the metastasis and nonmetastasis groups (Table S4). The proportion of participants with abnormal TM values stratified by pathology subtype is shown in Figure S3C,D. When stratified by pathology subtype according to the cut‐off value, the proportion of participants with abnormal CYFRA values was significantly different between the metastasis and nonmetastasis groups in NSCLC compared with that stratified by pathology subtype according to the URL (Figure S3C,D). The Cochran‐Mantel‐Haenszel test showed that the difference in the number distribution of each TM between the metastasis group and nonmetastasis group was not affected by the tumor subtypes (χ^2^ = 0.094, *P* = .760; Table S5).

### 3.4 Receiver operating characteristic curves

In the forest plot, age (under 63 years), gender, and biomarkers (except CA724) grouped by the cut‐off value were all independent high‐risk factors for metastasis (*P* < .05; Figure S4).

The logistic regression models to assess metastasis were established based on the combination of the levels of CEA, CYFRA, NSE, CA125, CA153, CA199, and CA724 (crude‐level), based on the combination of the binarization according to the URL range of a single biomarker (crude‐URL), and based on the combination of the binarization according to the cut‐off values of single biomarkers (crude cut‐off). On the basis of the three models mentioned above, logistic regression models with the addition of gender and age were also established, named comb‐level, comb‐URL, and comb‐cut‐off models. Stepwise regressions based on the seven TMs and the patients’ gender and age were established (named the step‐level, step‐URL, and step‐cut‐off models).

The results indicated that the comb‐cut‐off model had the highest AUC (0.792) and highest specificity (0.871; Figure 2A,B). The comb‐cut‐off alternative model was better than the logistic regression models combined and not combined with gender and age for single markers divided into two groups by the cut‐off value (Figure 2C,D). Similar results were observed in the comparison between logistic regression models combined and not combined with gender and age for single markers divided into two groups by the URL and the comb‐cut‐off alternative model (Figure S5A,B). These results suggested that the comb‐cut off model had the optimal AUC and sensitivity in assessing tumor metastasis in lung cancer. In addition, it showed that a single TM was not enough to exceed the combined TMs as the evaluation criteria.

### 3.5 Development and application of the clinical prediction model

**FIGURE 3:**
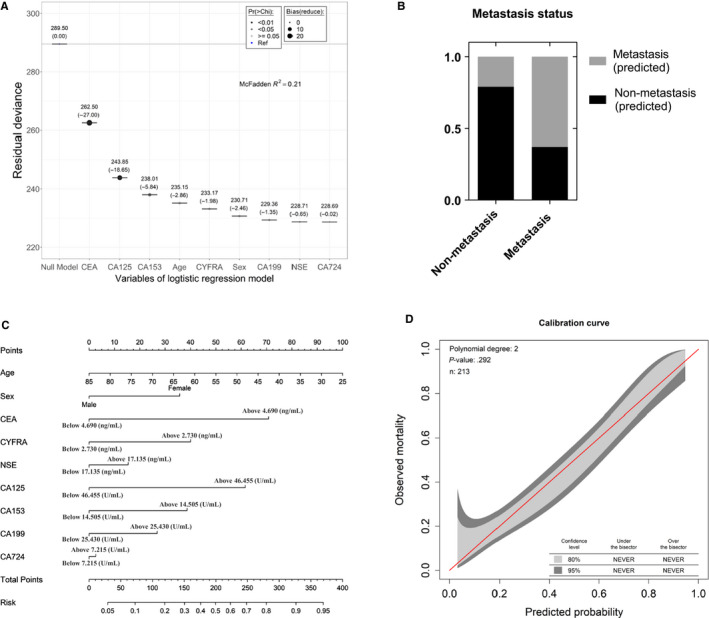
Development of the prediction model. A, Assessment for the model parameters. B, Comparison of actual results and predicted results. C, Nomogram of the regression model to predict metastasis. D, Calibration belt of the nomogram for the probability of lung cancer patients with metastasis

The difference between the null deviance and residual deviance of the alternative logistic regression model was assessed to evaluate the model parameters (Figure 3A). Carcinoembryonic antigen, CA125, and CA153 had a greater impact on the entire model, whereas NSE and CA724 had a small impact on the entire model. A total of 79% of the nonmetastasis patients and 63% of the metastasis patients could be recognized by the comb‐cut‐off model (Figure 3B). A nomogram of the regression model was used to visualize the results of the comb‐cut‐off model (Figure 3C). Four examples of assessing metastasis with the nomogram of the regression model with gender, age, and the seven TMs are shown in Figure S6A‐E. The total points were calculated by adding all scores of the age factor, gender factor, and TM factors. The odds scores were obtained by comparing the results of the total points on the odds line. The 95% CIs of GiViTI (Italian Group for the Evaluation of the Interventions in Intensive Care Units) calibration belt did not cross the diagonal bisector line, and the *P*‐value in GiViTI calibration test was .292 (Figure 3D). These results indicated that this model is a reliable tool for metastasis assessment and could be easily and conveniently applied in clinical practice.

**FIGURE 4:**
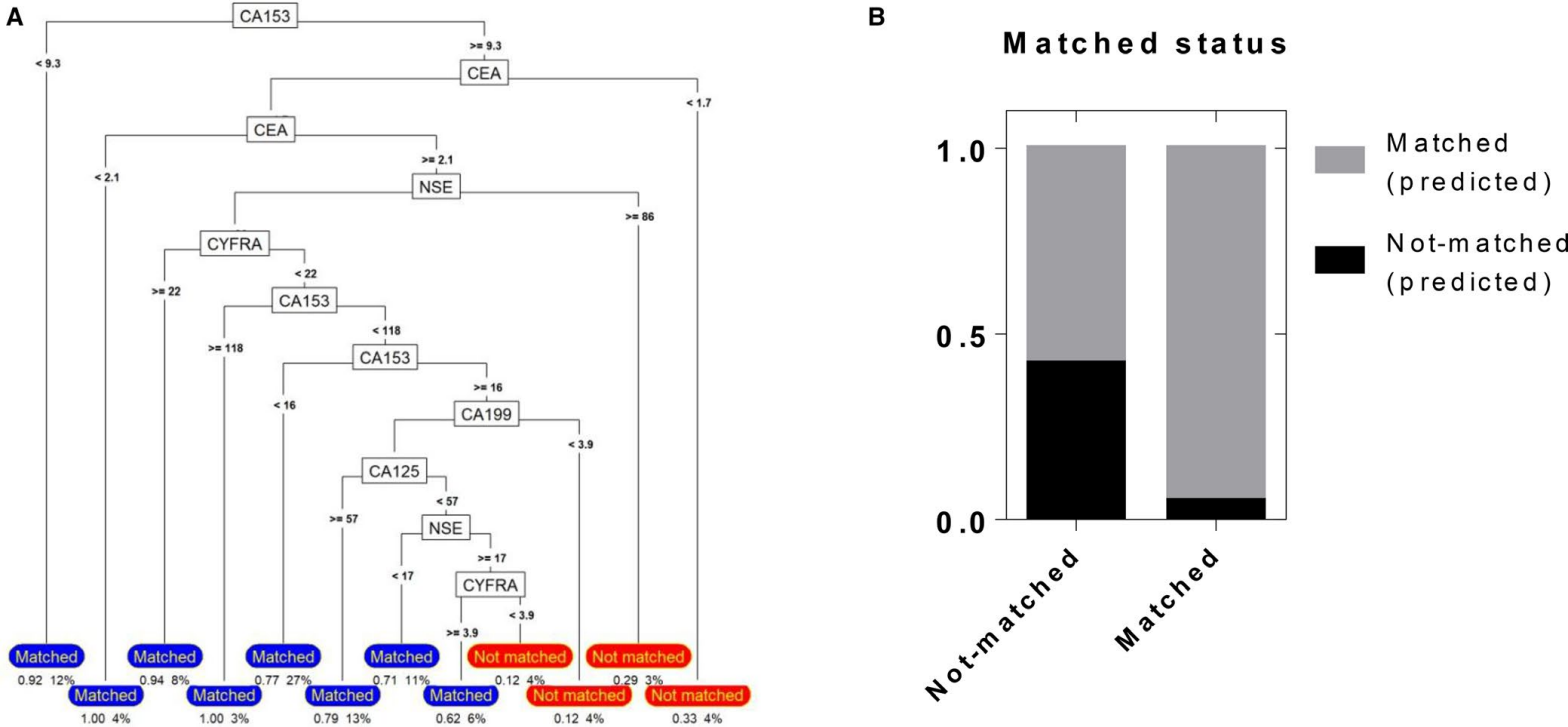
Performance of the decision tree model. A, The rules of the decision tree model, which was based on the levels of the individual biomarkers and the performance of the logistic regression model compared with the actual value. B, Performance of the decision tree model

To optimize the comb‐cut‐off model, a decision tree model was established based on the levels of the individual biomarkers and the performance of the logistic regression model compared with the actual value. The rules of the decision tree model are presented in Figure 4A. The matched results represented 79% of the nonmetastasis patients (true negatives) and 63% of the metastasis patients (true positives). The nonmatched results represented that the actual results of the patients were not in line with the predicted results. Before applying the comb‐cut‐off model, the applicability of the comb‐cut‐off model to patients was evaluated according to the decision tree. If the patients met the "Matched" condition, the comb‐cut‐off model could be used, and the predicted matched results could be up to 95% fit to the actual matched results (Figure 4B). However, if the patient meets the "Not matched" condition according to the decision tree model, more other clinical methods should be considered to detect metastasis. These results suggest that the comb‐cut‐off model and decision tree model should be combined to increase the accuracy of the prediction in the clinic.

## 4 DISCUSSION

In this retrospective study, we researched several serum biomarkers as different combinations for assessing tumor metastasis in newly diagnosed lung cancer patients. Patients with metastasis had significantly increased serum CEA, CA125, CA199, CA153, CYFRA, and NSE levels vs patients without metastasis. The ROC curve demonstrated that the CEA‐CA125‐CA199‐CA153‐CYFRA‐NSE‐CA724 combination based on the cut‐off value had good capability in assessing tumor metastasis. We combined gender, age, and the cut‐off value of seven TMs to construct the comb‐cut‐off model. Our data also showed that the comb‐cut‐off model was reliable in assessing lung cancer patients with or without metastasis. The decision tree model provided a certain guiding value for the clinical application of our model. These results suggest that the combined application of the decision tree model and the nomogram of the regression model in our study are convenient and successful in assessing tumor metastasis in newly diagnosed lung cancer patients.

Carcinoembryonic antigen, which was first found in 1965 in the blood of patients with colon cancer, is a serum glycoprotein and is the most common marker for many cancers. However, the application value of CEA in lung cancer is still controversial.^15^ Carbohydrate antigen is a kind of related antigen of cancer cells. The commonly used CA series are CA125, CA199, CA153, and CA724. They have a long history in the auxiliary diagnosis of tumors and usually have special glycan structures with moderate sensitivity and specificity.^16^ The standard reference values of the TMs were provided by a test kit as follows: CEA: 0‐5 ng/mL, CA125: 0‐35 U/mL, CYFRA: 0‐3.3 ng/mL, and NSE: 0‐16.3 ng/mL. The serum CA125 level was considered to be related to metastasis but not associated with a certain pathological type. A study reported that the CA125 levels in <25% of patients with metastasis were higher than 15 U/mL.^17^ In our study, even the mildest group (tumor size ≤ 3 cm) had a mean CA125 level of 26 U/mL, which was much higher than 15 U/mL. Therefore, the appropriate reference value should be further studied. The URL value has been chosen as the boundary in clinical cancer diagnosis. However, the URL value of some TMs could not successfully stratify patients according to the status of metastasis in our study. Hence, the cut‐off value of each individual TM was calculated by logistic regression based on each TM. We further compared the URL and cut‐off value to assess tumor metastasis. We found that the ROC curves had higher AUC values and specificity when the logistic regression models were based on the cut‐off values of the TMs. Although studies have reported that a single biomarker, such as CYFRA, can be used as the preferred marker for the prediction of lung cancer.^18^ Many studies pointed out that multiple serum TMs are more accurate in screening lung cancer than individual TMs.^19^, ^20^, ^21^, ^22^ Our results revealed that combined TMs based on cut‐off values showed better accuracy than single biomarkers in tumor metastasis assessment.

The odds ratio of age, gender, and the seven biomarkers grouped by the cut‐off value showed that these variables were all risk factors for metastasis. Females had a higher metastatic risk (OR = 1.90, 95% CI: 1.26‐2.88, *P* < .05) than males. Patients who were under 63 years old had a higher risk of tumor metastasis (OR = 1.67, 95% CI: 1.03‐2.70, *P* < .05), and the prospective risk of tumor metastasis increased with a decrease in age. A previous study found that bone metastasis was significantly increased in female mice than in male mice, while other sites were not significant.^23^ Gender, age, and tumor size have a complex relationship in resected NSCLC.^24^ These findings suggest that sex hormones and aging may be involved in the differentiation and spread of cancer cells, and it is important to pay more attention to the interactions among gender, age, and metastasis in lung cancer. CEA, CA125, and CA153 had the most influence on the model, while NSE and CA724 had minimum impact on the prediction model. The results showed that although the weights of each index are different, they could not be chosen subjectively. We found that incorporating the cut‐off value of the seven TMs and patients' age and gender showed the highest AUC and specificity in metastasis assessment. Therefore, the development of the prediction model was based on the combined cut‐off value of TMs and patients’ basic characteristics.

Metastases are routinely detected by surgeries and CT, PET, MRI, and PET‐CT scans. However, financial burdens and radiation could limit the use of these methods in the detection of metastasis to a certain extent. Our nomogram of the regression model was based on patients' gender, age, and TM levels in serum, which are accessible and convenient. In this study, we found the variation pattern of seven TMs in lung cancer metastasis. For instance, in NSCLC metastasis, the CA125‐CA153‐CA199‐CEA‐CYFRA‐NSE combination showed an increase simultaneously, while in SCLC, the CA199‐CEA combination increased. Therefore, if these TMs were found to be abnormally elevated in a certain pattern, we should be alarmed to the risk of metastasis and should use the model to judge the necessity of further examinations. Based on the deviation of the model, we trained the decision tree model to guide the rational application of the model. According to the rules in the decision tree, we can prejudge whether the patients belong to the matched or not matched groups. If patients are matched, the nomogram of the regression model can be further applied to assess metastasis. If patients are not matched, further decisions can be made based on the clinical characteristics. In patients highly suspected of lung cancer, we could predict metastasis with a combination of the nomogram of the regression model and the decision tree model.

The limitation of the study was that the model was not based on multicenter cohort data, which may make the results less generalizable. We have not yet studied the relationship between the TMs and the metastatic sites or pathological types of lung cancer, which requires more cases and refined models. Future studies on multicentric cohorts should include more patients and improve the generalizability of the prediction model.

## 5 CONCLUSIONS

Our study suggests that the combination of the prediction model with gender, age, and seven serum TMs and the decision tree model was efficient and reliable for assessing tumor metastasis before traditional standard methods are applied in highly suspected lung cancer patients and could provide some positive suggestions in the clinic.

## CONFLICT OF INTEREST

All authors declared no conflict of interest.

## AUTHOR CONTRIBUTIONS

Guarantor of the article: Fanxin Zeng, PhD; specific author contributions: Fanxin Zeng, Xiuqin Zhang, and Jiasi Wang designed the study. Tingjie Wang, Fanwei Zeng, Yanpeng Chu, Jie Li, and Liangli Sun collected the data. Jiasi Wang, Min Wu, Qianlai Chen, and Pingxi Wang did the statistical analysis. Yanpeng Chu and Jie Li wrote the manuscript. Jiasi Wang, Fanxin Zeng, and Xiuqin Zhang revised the manuscript. All authors reviewed the manuscript and approved the final draft.

## Supporting information

Table S1

Table S2

Table S3

Table S4

Table S5

Appendix S1

Appendix S2

## Data Availability

The data are not publicly available as the data also forms part of an ongoing study. Some data used in the study are available from the corresponding author by request.
