# Supplementary material for: Development of a prediction model with serum tumor markers to assess tumor metastasis in lung cancer
Source: Cancer Med. 2020 Jun 14;9(15):5436–45. doi: 10.1002/cam4.3184 (PMC7402813; doi:10.1002/cam4.3184)
Supplement: Supplementary file 1 — Table S1 [file CAM4-9-5436-s001.docx]

**Supplementary Table 1.** Upper reference limit and cut-off value of individual biomarker.

| **Biomarkers** | **Upper reference limit** | **Cut-off value** | **Unit** |
| --- | --- | --- | --- |
| CA125 | 35.000 | 46.455 | U/mL |
| CA153 | 25.000 | 14.505 | U/mL |
| CA199 | 27.000 | 25.430 | U/mL |
| CA724 | 6.900 | 7.215 | U/mL |
| CEA | 5.000 | 4.690 | ng/mL |
| CYFRA | 3.300 | 2.730 | ng/mL |
| NSE | 16.300 | 17.135 | ng/mL |

Abbreviations: CA125, carbohydrate antigen 125 (U/mL); CA153, carbohydrate antigen 153 (U/mL); CA199, carbohydrate antigen 199 (U/mL); CA724, carbohydrate antigen 724 (U/mL); CEA, carcinoembryonic antigen (ng/mL); CYFRA , cytokeratin-19 fragment (ng/mL); NSE, neuron-specific enolase (ng/mL).
