# Supplementary material for: Development of a prediction model with serum tumor markers to assess tumor metastasis in lung cancer
Source: Cancer Med. 2020 Jun 14;9(15):5436–45. doi: 10.1002/cam4.3184 (PMC7402813; doi:10.1002/cam4.3184)
Supplement: Supplementary file 2 — Table S2 [file CAM4-9-5436-s002.docx]

**Supplementary Table 2.** Tumor markers values stratified by tumor size (Metastasis *versus* Non-metastasis).

| **Biomarkers** | **Tumor size**  **<= 3.0 cm** | **Tumor size**  **3.1-5.0 cm** | **Tumor size**  **5.1-7.0 cm** | **Tumor size**  **> 7.0 cm** |
| --- | --- | --- | --- | --- |
| CA125 |  |  |  |  |
| Non-metastasis | 18.0 (12.1-27.2) | 20.5 (12.9-50.1) | 35.2 (16.5-60.0) | 41.6 (24.9-90.1) |
| Metastasis | 26.1 (19.4-122.4)* | 44.1(18.0-83.3)* | 70.1 (39.2-245.1)* | 74.8 (43.5-142.7) |
| CA153 |  |  |  |  |
| Non-metastasis | 12.6 (9.9-21.9) | 13.8 (9.5-21.4) | 14.5 (10.2-20.7) | 18.4 (11.4-33.1) |
| Metastasis | 19.5 (13.4-38.2)* | 18.8 (14.6-37.1)* | 19.9 (12.2-41.6)* | 14.9 (11.9-25.6) |
| CA199 |  |  |  |  |
| Non-metastasis | 11.4 (8.1-18.0) | 8.8 (5.9-17.2) | 10.1 (7.0-21.9) | 10.6 (6.1-18.0) |
| Metastasis | 12.8 (9.2-34.8) | 20.2 (6.5-49.6)* | 15.0 (7.9-42.9) | 9.6 (5.8-21.1) |
| CA724 |  |  |  |  |
| Non-metastasis | 2.6 (1.2-5.2) | 4.0 (1.7-12.3) | 2.2 (1.4-6.0) | 6.9 (0.9-10.6) |
| Metastasis | 4.0 (1.8-9.9) | 8.6 (3.3-14.8) | 14.2 (3.6-15.8)* | 2.2 (1.7-5.3) |
| CEA |  |  |  |  |
| Non-metastasis | 3.0 (2.1-4.3) | 3.6 (2.8-5.5) | 3.4 (2.5-4.3) | 3.7 (2.3-6.4) |
| Metastasis | 10.4 (4.0-60.5)* | 6.9 (3.1-24.0)* | 7.6 (4.3-31.2)* | 7.0 (2.3-12.5) |
| CYFRA |  |  |  |  |
| Non-metastasis | 3.4 (2.4-5.1) | 3.8 (2.5-7.5) | 6.0 (3.7-11.5) | 9.0 (4.3-13.9) |
| Metastasis | 5.2 (2.7-9.4) | 4.5 (3.0-7.5) | 7.6 (4.9-23.4) | 8.2 (5.0-12.2) |
| NSE |  |  |  |  |
| Non-metastasis | 13.8 (9.9-22.7) | 14.5 (10.7-19.6) | 19.6 (15.3-29.5) | 23.1 (16.8-30.5) |
| Metastasis | 18.1 (12.9-22.9) | 14.5 (11.4-23.7) | 22.1 (15.4-29.5) | 41.5 (15.5-128.2) |

Abbreviations: CA125, carbohydrate antigen 125 (U/mL); CA153, carbohydrate antigen 153 (U/mL); CA199, carbohydrate antigen 199 (U/mL); CA724, carbohydrate antigen 724 (U/mL); CEA, carcinoembryonic antigen (ng/mL); CYFRA, cytokeratin-19 fragment (ng/mL); NSE, neuron-specific enolase (ng/mL)*.*

Data are given as median (IQR).

**, p< 0.05, Non-metastasis versus Metastasis.*
