# Supplementary material for: Development of a prediction model with serum tumor markers to assess tumor metastasis in lung cancer
Source: Cancer Med. 2020 Jun 14;9(15):5436–45. doi: 10.1002/cam4.3184 (PMC7402813; doi:10.1002/cam4.3184)
Supplement: Supplementary file 3 — Table S3 [file CAM4-9-5436-s003.docx]

**Supplementary Table 3.** Numbers stratified by tumor size (Metastasis *versus* Non-metastasis).

| **Biomarkers** | **Tumor size**  **<= 3.0 cm** | **Tumor size**  **3.1-5.0 cm** | **Tumor size**  **5.1-7.0 cm** | **Tumor size**  **> 7.0 cm** |
| --- | --- | --- | --- | --- |
| **CA125** | | | | |
| Non-metastasis | 43 | 73 | 50 | 26 |
| Metastasis | 33 | 62 | 41 | 24 |
| **CA153** | | | | |
| Non-metastasis | 39 | 66 | 45 | 21 |
| Metastasis | 33 | 57 | 37 | 20 |
| **CA199** | | | | |
| Non-metastasis | 44 | 74 | 51 | 26 |
| Metastasis | 34 | 62 | 40 | 22 |
| **CA724** | | | | |
| Non-metastasis | 25 | 40 | 23 | 13 |
| Metastasis | 17 | 27 | 13 | 9 |
| **CEA** | | | | |
| Non-metastasis | 45 | 76 | 54 | 28 |
| Metastasis | 39 | 66 | 42 | 24 |
| **CYFRA** | | | | |
| Non-metastasis | 29 | 46 | 28 | 15 |
| Metastasis | 18 | 36 | 19 | 10 |
| **NSE** | | | | |
| Non-metastasis | 30 | 46 | 28 | 17 |
| Metastasis | 18 | 35 | 21 | 9 |

Abbreviations: CA125, carbohydrate antigen 125 (U/mL); CA153, carbohydrate antigen 153 (U/mL); CA199, carbohydrate antigen 199 (U/mL); CA724, carbohydrate antigen 724 (U/mL); CEA, carcinoembryonic antigen (ng/mL); CYFRA, cytokeratin-19 fragment (ng/mL); NSE, neuron-specific enolase (ng/mL).

*Cochran-Mantel-Haenszel M^2^ = 0.708, p* = 0.87.
