# Supplementary material for: Development of a prediction model with serum tumor markers to assess tumor metastasis in lung cancer
Source: Cancer Med. 2020 Jun 14;9(15):5436–45. doi: 10.1002/cam4.3184 (PMC7402813; doi:10.1002/cam4.3184)
Supplement: Supplementary file 4 — Table S4 [file CAM4-9-5436-s004.docx]

**Supplementary Table 4.** Tumor markers values stratified by pathology subtype (Metastasis *versus* Non-metastasis).

| **Biomarkers** | **NSCLC** | |  | **SCLC** | |
| --- | --- | --- | --- | --- | --- |
|  | **Metastasis** | **Non-metastasis** |  | **Metastasis** | **Non-metastasis** |
| CA125 | 61.6 (26.1-155.0)* | 26.2 (14.7-53.2) |  | 57.8 (22.5-148.9) | 38.3 (18.0-78.8) |
| CA153 | 21.2 (13.6-44.1)* | 13.7 (9.8-21.6) |  | 16.3 (12.2-24.3) | 16.7 (12.1-21.5) |
| CA199 | 14.9 (7.0-59.0)* | 10.4 (6.4-17.1) |  | 15.5 (9.0-38.9)* | 11.1 (6.3-18.5) |
| CA724 | 5.6 (1.9-16.4) | 4.5 (1.7-11.3) |  | 3.7 (0.9-9.9) | 2.2 (1.0-14.5) |
| CEA | 7.5 (3.5-27.0)* | 3.4 (2.5-5.1) |  | 7.2 (3.2-25.9)* | 3.6 (2.1-6.2) |
| CYFRA | 7.1 (3.6-16.9)* | 5.5 (2.9-10.1) |  | 4.2 (2.9-6.0) | 4.4 (2.6-5.9) |
| NSE | 17.0 (12.8-23.6)* | 14.3 (10.6-19.1) |  | 42.7 (24.9-128.2) | 36.6 (26.6-60.5) |

Abbreviations: NSCLC, non-small cell lung cancer; SCLC, small cell lung cancer; CA125, carbohydrate antigen 125 (U/mL); CA153, carbohydrate antigen 153 (U/mL); CA199, carbohydrate antigen 199 (U/mL); CA724, carbohydrate antigen 724 (U/mL); CEA, carcinoembryonic antigen (ng/mL); CYFRA, cytokeratin-19 fragment (ng/mL); NSE, neuron-specific enolase (ng/mL)*.*

Data are given as median (IQR).

**, p< 0.05, Non-metastasis versus Metastasis.*
