# Supplementary material for: Development of a prediction model with serum tumor markers to assess tumor metastasis in lung cancer
Source: Cancer Med. 2020 Jun 14;9(15):5436–45. doi: 10.1002/cam4.3184 (PMC7402813; doi:10.1002/cam4.3184)
Supplement: Supplementary file 5 — Table S5 [file CAM4-9-5436-s005.docx]

**Supplementary Table 5.** Numbers stratified by pathology subtypes (Metastasis *versus* Non-metastasis).

| **Biomarkers** | **NSCLC** | |  | **SCLC** | |
| --- | --- | --- | --- | --- | --- |
|  | **Metastasis** | **Non-metastasis** |  | **Metastasis** | **Non-metastasis** |
| CA125 | 169 | 203 |  | 61 | 52 |
| CA153 | 154 | 183 |  | 60 | 52 |
| CA199 | 170 | 204 |  | 60 | 54 |
| CA724 | 73 | 111 |  | 22 | 25 |
| CEA | 185 | 214 |  | 63 | 55 |
| CYFRA | 90 | 131 |  | 25 | 28 |
| NSE | 89 | 131 |  | 26 | 30 |

Abbreviations: CA125, carbohydrate antigen 125 (U/mL); CA153, carbohydrate antigen 153 (U/mL); CA199, carbohydrate antigen 199 (U/mL); CA724, carbohydrate antigen 724 (U/mL); CEA, carcinoembryonic antigen (ng/mL); CYFRA , cytokeratin-19 fragment (ng/mL); NSE, neuron-specific enolase (ng/mL).

*Mantel-Haenszel X^2^ = 0.094, p = 0.760. Patients with unknow pathology subtype were excluded in the difference analysis (n = 20).*
