# Supplementary material for: Development of a prediction model with serum tumor markers to assess tumor metastasis in lung cancer
Source: Cancer Med. 2020 Jun 14;9(15):5436–45. doi: 10.1002/cam4.3184 (PMC7402813; doi:10.1002/cam4.3184)
Supplement: Supplementary file 6 — Appendix S1 [file CAM4-9-5436-s006.docx]

**Appendix 1**

1. **Tumor Biomarker Assays**

Serum CEA (standard reference range: 0-5 ng/mL), CA125 (standard reference range: 0-35 U/mL), CA153 (standard reference range: 0-25 U/mL), CA199 (standard reference range: 0-27 U/mL), CA724 (standard reference range: 0-6.9 U/mL), CYFRA (standard reference range: 0-3.3 ng/mL), and NSE (standard reference range: 0-16.3 ng/mL) levels were detected at the admission according to the manufacturer’s instructions. The experimental operations were carried out according to the reagent protocols. All kits used to detect the above TMs were obtained from Roche and tested on a Roche E601 system.

1. **Statistical Analysis**

The results are expressed as numbers, medians (with interquartile ranges, IQRs) or proportions. The Wilcoxon test was used to compare the differences in the levels of the TMs, and *t*-test was used to compare the differences in age. A chi-square test was used to compare the proportions between groups. The independence test of categorical variables was based on the chi-square independence test or the Mantel-Haenszel test. Receiver operating characteristic (ROC) curves were calculated for logistic regressions based on a single biomarker or multiple biomarkers (and/or combined sex and age) and stepwise regressions in which the mode of stepwise search was used. Whether the Akaike's information criterion (AIC) value for a stepwise regression incorporating multiple variables was significantly decreased was observed. The variance inflation factor (VIF) was used to judge whether there was multicollinearity in logistic regression based on multiple markers, and VIF > 2indicated the existence of multicollinearity. There was no multicollinearity in all logistic regression models based on multiple markers in this study. The pROC package (1.14.0) was used for the ROC calculations and comparisons [^1^](#_ENREF_1). Delong's test was used to detect differences between two ROC curves. If *p*-value > 0.05 when comparing two ROC curves, the ROC curve with the larger area under the curve (AUC) value and the higher specificity was the better choice. A decision tree model was constructed to guide the clinically rational application of the nomogram model. R (version: R 3.4.3 for Windows (x64), https://www.r-project.org/) was used for statistical analysis. A *p*-value < 0.05 was considered statistically significant.

**References**

1. Robin X, Turck N, Hainard A, et al. pROC: an open-source package for R and S+ to analyze and compare ROC curves. BMC Bioinformatics. 2011;12: 77.
