# Supplementary material for: Development of a prediction model with serum tumor markers to assess tumor metastasis in lung cancer
Source: Cancer Med. 2020 Jun 14;9(15):5436–45. doi: 10.1002/cam4.3184 (PMC7402813; doi:10.1002/cam4.3184)
Supplement: Supplementary file 7 — Appendix S2 [file CAM4-9-5436-s007.pdf]

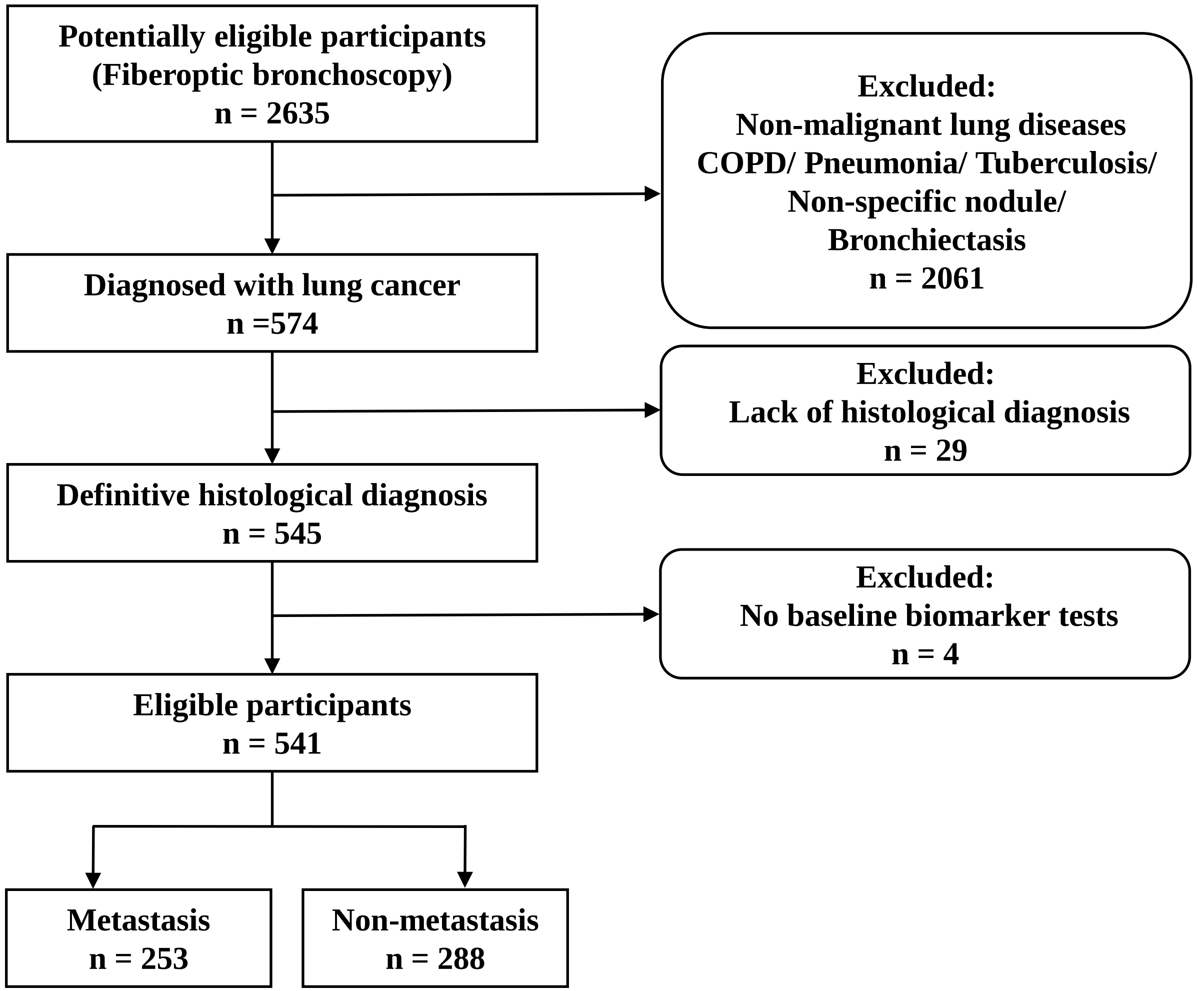

Supplementary Figure 1. Enrollment of patients.

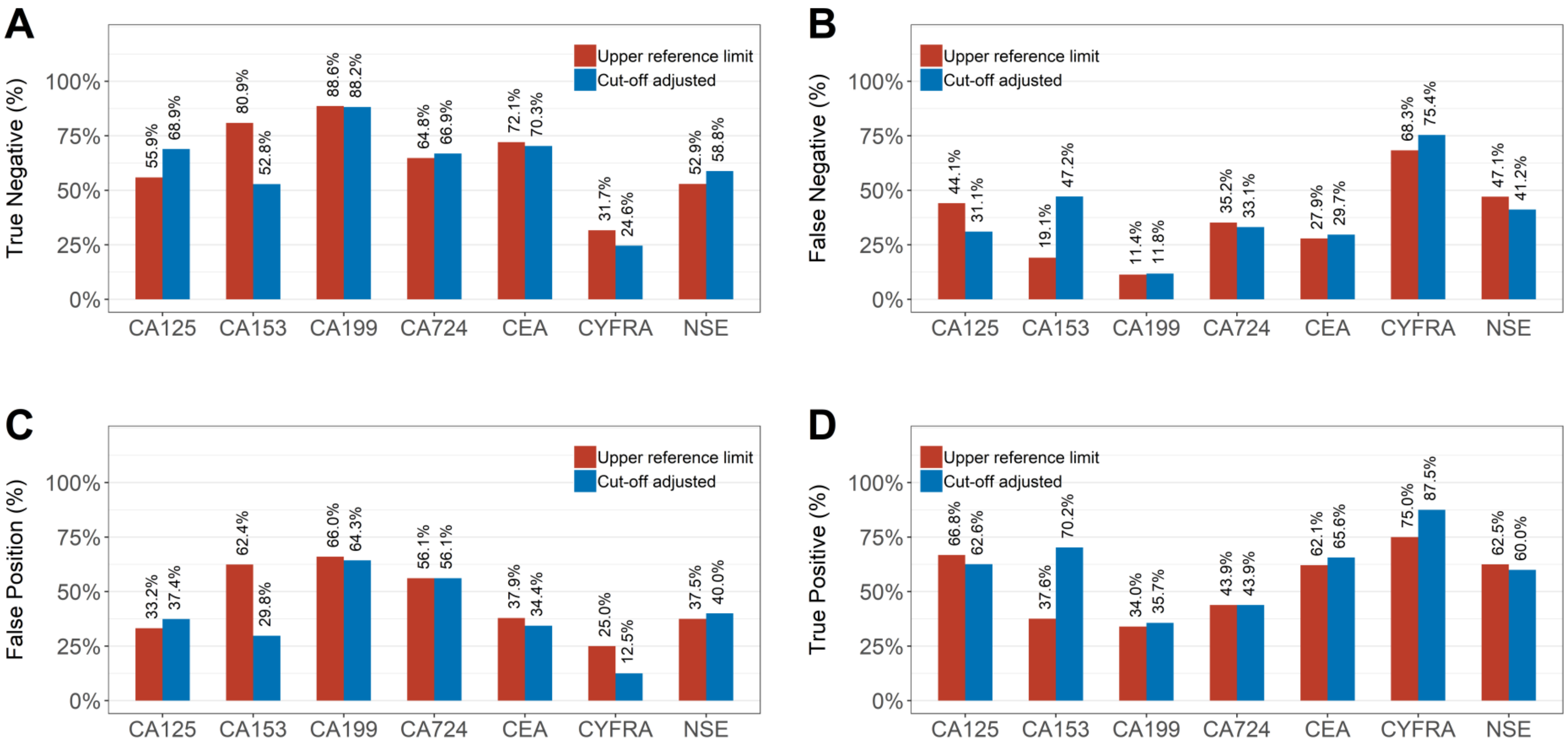

Supplementary Figure 2. TNR, FNR, FPR and TPR of each individual TM investigated assessment. (A) Difference of TNR(True Negative Rate), (B) FNR(False Negative Rate), (C) FPR(False Positive Rate) and (D) TPR(True Negative Rate) in different grouping methods (Group by upper reference limits versus Group by cut-off value of logistic regression, single biomarker).

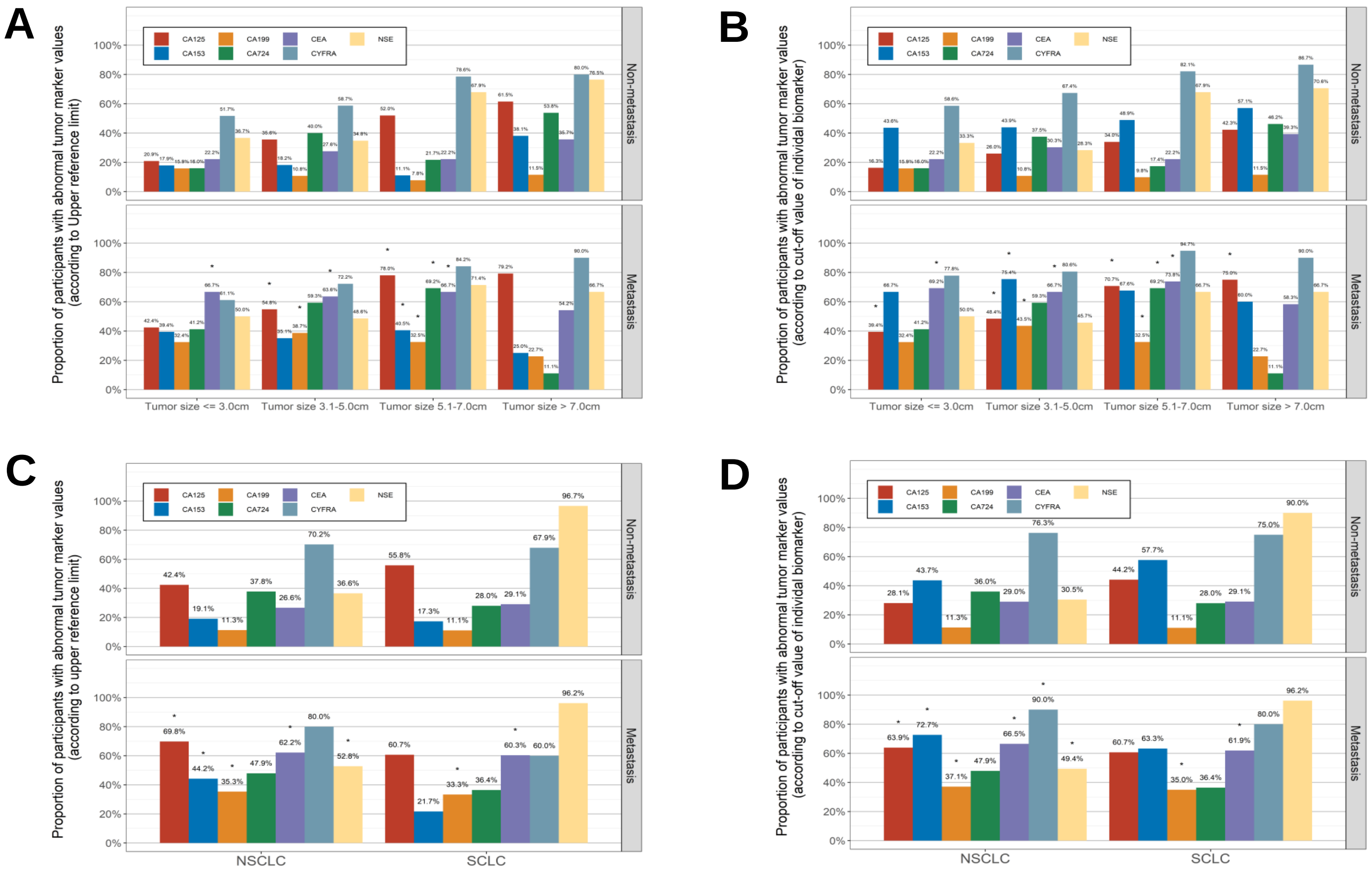

Supplementary Figure 3. Proportion of participants with abnormal tumor marker values stratified by nodule size and pathology subtype. (A) stratified by nodule size according to upper reference limit. (B) stratified by nodule size according to cut-off value. (C) stratified by pathology subtype according to upper reference limit. (D) stratified by pathology subtype according to cut-off value.

Abbreviations: NSCLC, non-small cell lung cancer; SCLC, small cell lung cancer; CA125, carbohydrate antigen 125 (U/mL); CA199, carbohydrate antigen 199 (U/mL); CEA, carcinoembryonic antigen (ng/mL); NSE, neuron-specific enolase (ng/mL); CA153, carbohydrate antigen 153 (U/mL); CA724, carbohydrate antigen 724 (U/mL); CYFRA , cytokeratin-19 fragment (ng/mL). *, $p < 0.05$, Non-metastasis *versus* Metastasis.

	Groups	Metastasis	Non Metastasis	OR (95%CI)	p-value
Sex	Male	182	239	1(ref)	
	Female	71	49	1.90(1.26-2.88)	0.002
Age	(68,83]	50	80	1(ref)	
	(63,68]	52	70	1.19(0.72-1.97)	0.501
	(54,63]	75	72	1.67(1.03-2.70)	0.037
	[26,54]	76	66	1.84(1.14-3.00)	0.013
CA125	Below	88	186	1(ref)	
	Above	147	84	3.70(2.57-5.37)	<0.001
CA153	Below	65	130	1(ref)	
	Above	153	116	2.64(1.80-3.88)	<0.001
CA199	Below	151	240	1(ref)	
	Above	84	32	4.17(2.67-6.65)	<0.001
CA724	Below	55	95	1(ref)	
	Above	43	47	1.58(0.93-2.69)	0.091
CEA	Below	87	199	1(ref)	
	Above	166	84	4.52(3.15-6.53)	<0.001
CYFRA	Below	15	41	1(ref)	
	Above	105	126	2.28(1.22-4.46)	0.012
NSE	Below	48	100	1(ref)	
	Above	72	70	2.14(1.34-3.46)	0.002

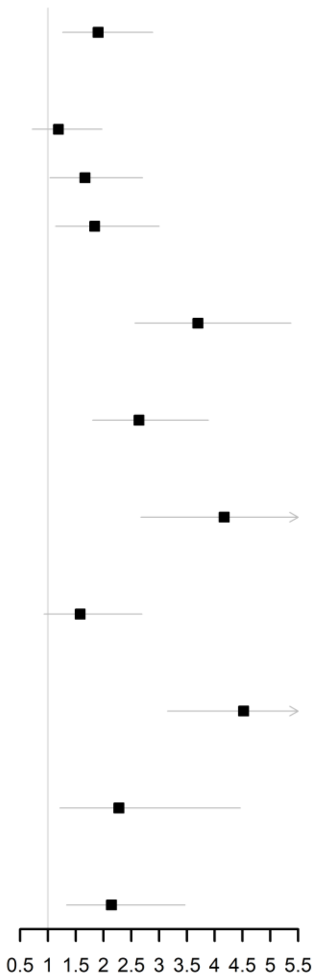

Supplementary Figure 4. Odds ratio of Age, Sex and biomarkers grouped by cut-off value. Below: below cut-off value. Above: above cut-off value.

Abbreviations: CA125, carbohydrate antigen 125 (U/mL); CA153, carbohydrate antigen 153 (U/mL); CA199, carbohydrate antigen 199 (U/mL); CA724, carbohydrate antigen 724 (U/mL); CEA, carcinoembryonic antigen (ng/mL); CYFRA , cytokeratin-19 fragment (ng/mL); NSE, neuron-specific enolase (ng/mL).

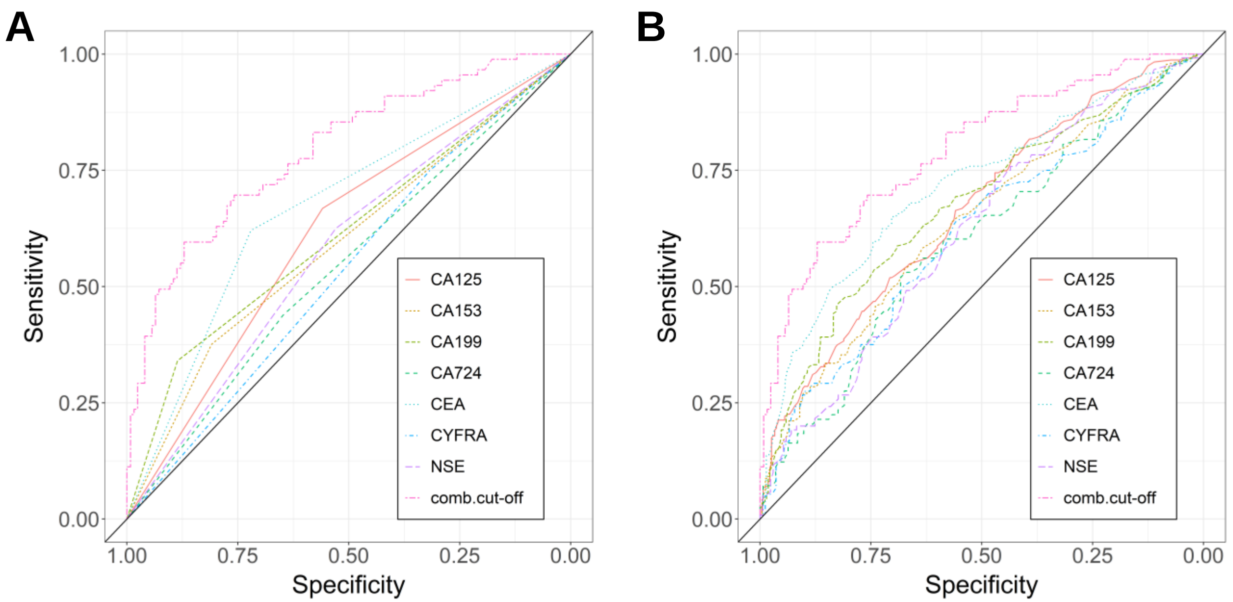

Supplementary Figure 5. Comparison alternative logistic model with models based on upper reference limit. (A) Adjusted without sex and age factors. (B) Adjusted with sex and age factors.

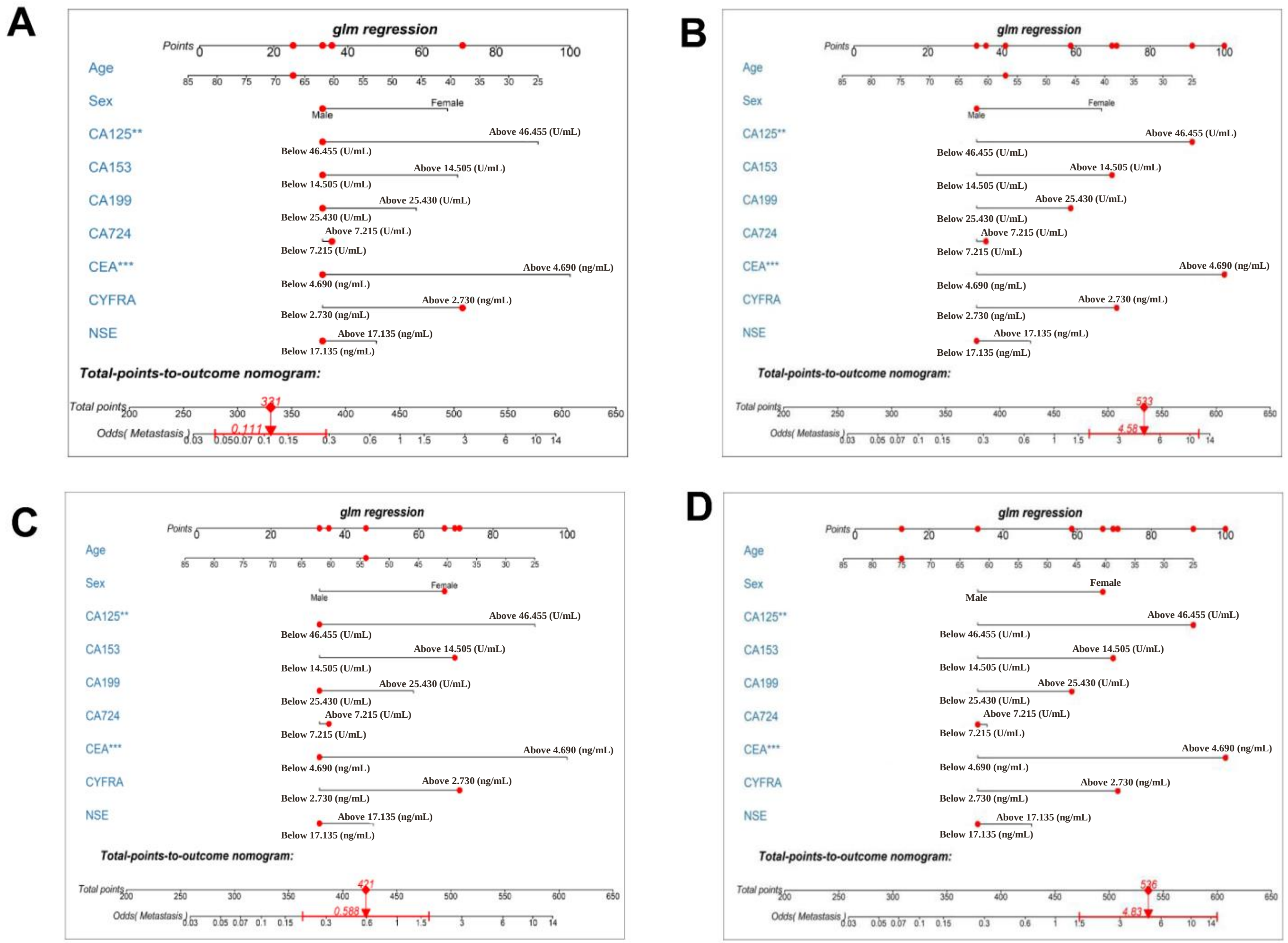

E

Sex	Age	CA125	CA153	CA199	CA724	CEA	CYFRA	NSE	Score	Predicted status	Actual status	No. Fig
Male	67	36.97	8.36	7.62	116.50	2.43	7.63	8.85	0.11	Non-Metastasis	Non-Metastasis	A
Male	57	70.88	18.64	26.43	15.46	4.91	6.34	8.96	4.58	Metastasis	Metastasis	B
Female	54	22.51	37.75	10.62	10.85	0.59	15.44	12.51	0.59	Non-Metastasis	Non-Metastasis	C
Female	75	351.00	26.54	354.10	0.70	81.33	6.58	16.99	4.83	Metastasis	Metastasis	D

Supplementary Figure 6. Four examples for nomogram model application.
